# Molecular Insights Into Canine Hepatocellular Carcinoma: A Cross‐Species Transcriptomic Comparison With Human HCC

**DOI:** 10.1002/mc.70092

**Published:** 2026-02-10

**Authors:** Mohammad Arif, MD Nazmul Hasan, Nobuhiro Nozaki, Yutaro Ide, Yoshiyuki Akiyama, Shaohsu Wang, Most Shumi Akhter Shathi, Osamu Yamato, Naoki Miura

**Affiliations:** ^1^ Joint Graduate School of Veterinary Medicine Kagoshima University Kagoshima Japan; ^2^ Department of Microbiology and Hygiene Bangladesh Agricultural University Mymensingh Bangladesh; ^3^ Veterinary Teaching Hospital, Joint Faculty of Veterinary Medicine Kagoshima University Kagoshima Japan

**Keywords:** bioinformatics, biomarker discovery, comparative genomics, comparative oncology, cross‐species transcriptomics, hepatocellular carcinoma, next‐generation sequencing (NGS), oncogenic pathways, transcriptome profiling

## Abstract

Canine hepatocellular carcinoma (HCC) requires further molecular characterization to identify diagnostic and therapeutic targets, and to establish whether dogs with this condition can model the human disease. Accordingly, we aimed to identify differentially expressed genes (DEGs) in canine HCC and evaluate cross‐species transcriptomic dysregulation in canine and human HCC. Liver tissue samples from three dogs with HCC and three healthy dogs were subjected to next‐generation sequencing, followed by RT‐qPCR validation. Identified DEGs were then targeted in bioinformatics analyses (pathway enrichment, protein‐protein interaction network, and hub gene analyses) for molecular characterization and comparison with human HCC datasets. We identified 975 DEGs (upregulated: 604; and downregulated: 371). Extracellular matrix‐receptor interaction, focal adhesion, cell adhesion molecule, PI3K/Akt signaling, and cytokine/chemokine‐related pathways were enriched. C1R, APOC3, C1QA, APOA1, C1QB, ACTG1, C1QC, CRP, ANXA5, and ANXA2 were identified as hub genes. Canine and human HCCs share 118 DEGs, highlighting conserved alterations in metabolic pathways, PI3K‐Akt signaling, focal adhesion, and PPAR signaling pathways. Based on human HCC data, SPP1, NQO1, RRM2, APOA1, APOC3, ALDOB, and IGF1 were identified as prognosticators indicating poor overall survival. This study presents the first cross‐species transcriptomic analysis of canine HCC, revealing significant molecular resemblances to human HCC, indicating it may be a promising comparative model for studying tumor biology, drug responses, and novel therapeutic interventions.

## Graphical abstract

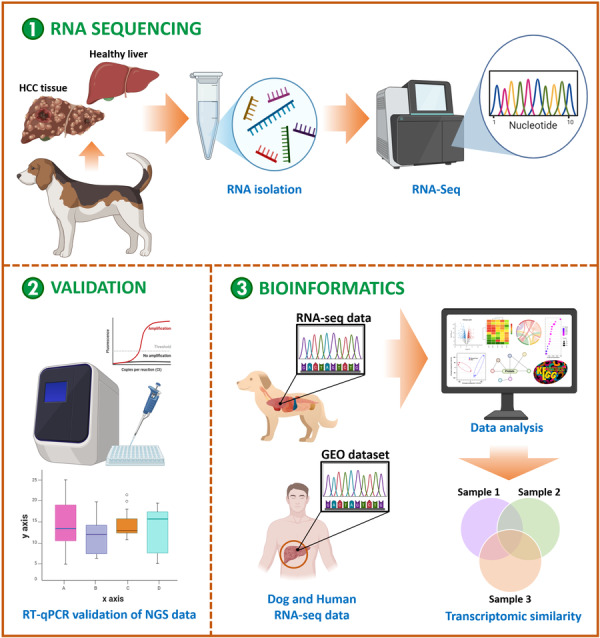

**Abbreviations**
ABCB4: ATP binding cassette subfamily B member 4
APOA1: apolipoprote1
DAVID: the database for annotation visualization and integrated discovery
DEGs: differentially expressed genes
ECM: extracellular matrix
EMT: epithelial‐mesenchymal transition
ENCORI: the encyclopedia of RNA interactomes
FDR: false discovery rate
GAPDH: glyceraldehyde 3‐phosphate dehydrogenase
GEO: gene expression omnibus
GEPIA: gene expression profiling interactive analysis
GO: gene ontology
HCC: hepatocellular carcinoma
KEGG: kyoto encyclopedia of genes and genomes
KM: kaplan‐meier
KUVTH: Kagoshima university veterinary teaching hospital
logFC: log₂ fold change
MCC: maximal clique centrality
MCODE: molecular complex detection
OR: odds ratio
OS: overall survival
PCA: principal component analysis
RR: relative risk
STRING: search tool for the retrieval of interacting genes/proteins
TCGA: the cancer genome atlas
TPM: transcripts per million

## 1 Introduction

Canine hepatocellular carcinoma (HCC) may partially mimic human HCC [1, 2]. Although etiology (viral infection and cirrhosis are not common triggers in dogs) and prevalence (relatively low in dogs) reportedly differ between human and canine liver cancers [3, 4], the many common features are of oncological interest. In both species, age at onset is similar (adjusting for life span) and male sex is also a predisposing factor [5]. Furthermore, human and canine HCCs have the same histotypes (with the trabecular pattern as the most common), show immunohistochemical similarities (in staining for epidermal and vascular endothelial growth factor receptors), and metastasize at a similar rate and to similar sites [1, 6]. Based on the phenotypic and immunohistochemical similarities, it is feasible that canine and human HCCs have some common underlying tumor biology, and that advances in research in dogs may ultimately benefit human patients as well.

Generally, there are a number of reasons why the dog may be a good translational model in oncology research. Naturally occurring canine cancers provide distinct biological and translational advantages due to their shared tumor biology with humans. Dogs develop cancer spontaneously, within an intact immune system, and under environmental conditions similar to those experienced by humans [7], unlike many murine models, where tumors are artificially induced and fail to reflect tumor‐ microenvironment interactions [7] fully. Additionally, the high genomic and molecular homology between dogs and humans [8], along with shared oncogenic pathways and expression profiles [9], enhances translational relevance [10]. Companion dog clinical trials also generate realistic data on pharmacokinetics, toxicity, and therapeutic efficacy in genetically diverse tumors, offering a more predictive bridge to human applications [11].

In considering how translational research may be developed in HCC, it is worth noting that both dogs and humans tend to be diagnosed with this disease at a late stage and have a poor prognosis [12, 13]. Thus, identifying biomarkers for earlier diagnosis would fulfill a major unmet need for both species. Comparative transcriptomic analysis enables the prioritization of biologically and clinically relevant genes conserved across species, thereby strengthening confidence that these candidates represent actual driver events rather than species‐specific changes [14]. Such conserved alterations tend to be more translationally relevant, as they reflect fundamental tumor biology shared across mammalian liver cancers, and may reveal potential biomarkers and possibly even therapeutic targets with substantial translational value. Molecular biomarkers with potential translational benefit for human cancers have been identified in canine mammary gland tumors, osteosarcoma, and prostate cancer [15, 16, 17]. In contrast, the molecular landscape of canine hepatocellular carcinoma (HCC) remains comparatively underexplored. In human HCC, numerous oncogenic pathways including the p53, Wnt/β‐Catenin, EGFR, IGF, HGF/c‐MET, TGF‐B, Nf‐κB, VEGF, and Hippo pathways are well‐characterized and clinically relevant [18]. Importantly, these pathways are highly conserved in dogs, which suggests the possibility that dogs and human may share molecular diagnostic biomarkers. Furthermore, a conserved cross‐species landscape for human and canine HCC and the presence of common cell types suggested common cellular communication, and the potential for studies of canine HCC to reveal further cross‐species commonalities in the field of multi‐omics [19]. Oncologists may thus reasonably expect meaningful molecular commonalities between canine and human HCCs. The promising response of dogs to off‐label sorafenib therapy further supports the likelihood of shared oncogenic pathways between the two species [20, 21].

RNA sequencing enables the identification of differentially expressed genes (DEGs) associated with oncogenic activity [22], and such transcriptomic analyses have been reported for a range of human and veterinary cancers [23, 24, 25, 26]. However, there is a paucity of data on DEGs in canine HCC, with the sole previous report limited to orthologs of genes targeted in human HCC therapy [27]. Molecular mechanisms underlying canine HCC have remained largely unelucidated, as have DEG‐related pathways, and the correspondence between DEGs in canine and human HCCs. Furthermore, pathway and protein interaction analyses are needed to illuminate how aberrant gene expression drives oncogenic processes, to advance knowledge of cross‐species transcriptomes [23, 28, 29, 30]. Additionally, studies leveraging comparative transcriptomics between canine and human HCC remain scarce.

To address this gap, we set out to investigate DEGs in canine HCC and their orthologs in human HCC in a two‐stage comparative analytical study. The first stage involved the identification of DEGs, through comparison of clinical samples from dogs with HCC and healthy liver tissues, followed by pathway and protein interaction analyses. In the second stage, we subjected the identified DEGs to a dual‐species transcriptome analysis to identify common cancer‐specific pathways and obtain molecular insights that may advance diagnosis and treatment of HCC in both dogs and humans.

## 2 Materials and Methods

### 2.1 Clinical Samples

Liver tissues were collected from 16 dogs undergoing tumor resection for histopathologically confirmed HCC at Kagoshima University Veterinary Teaching Hospital (KUVTH) or affiliated clinics between September 2012 and July 2023, with owner consent (Table S1). The study population consisted of dogs from a range of breeds (Beagle, Shiba Inu, Miniature Schnauzer, Toy Poodle, Yorkshire Terrier, Shetland Sheepdog, Golden Retriever, and Crossbreeds), aged 10 to 14 years, with both sexes represented. Due to the ethical restrictions and hospital regulations, tumor‐adjacent healthy liver tissues could not be obtained from HCC cases. Therefore, liver tissues from six clinically healthy adult laboratory Beagle dogs (*n* = 6) were used as controls [31]. These animals were maintained under controlled laboratory conditions for unrelated research purposes at Shin Nippon Biomedical Laboratories Ltd. (Tokyo, Japan), and were humanly sacrificed in accordance with standard procedures. Liver tissues were aseptically collected from control dogs at necropsy, immediately preserved in RNAlater, transported under temperature‐controlled conditions to Kagoshima University, and stored at −80°C for molecular analysis. This study was approved by the Ethics Committee of Kagoshima University (Approval No. KVH220001).

### 2.2 RNA Extraction and Sequencing

Total RNA was extracted using the mirVana™ miRNA Isolation Kit (Thermo Fisher Scientific, Vilnius, Lithuania), quantified with a NanoDrop 2000c (Thermo Fisher Scientific Inc., USA), and assessed for integrity (RIN > 8) using an Agilent 2100 Bioanalyzer (Agilent Technologies, USA). RNA from six samples (HCC = 3, normal liver = 3) was sequenced using the NovaSeq. 6000 platform (Illumina) at Hokkaido System Science Co. Ltd., Japan, as described previously [23].

### 2.3 Bioinformatics Analysis

FASTQ‐formatted sequencing data were analyzed using CLC Genomics Workbench v24.0 (Qiagen, Germany). Reads were trimmed, quality‐checked, and mapped to the CanFam3.1 genome (NCBI) using annotated gene and mRNA tracks. Gene expression was normalized to transcripts per million (TPM). DEGs were identified by comparison between HCC and normal liver tissues, using a negative binomial model. Genes were filtered using the following criteria: |log_2_ fold change | > 1.5, *p* < 0.05, and maximum group mean (TPM) > 5. PCA, volcano, and heatmap plots were generated in CLC.

### 2.4 Quantitative Real‐Time PCR (RT‐qPCR) Analysis

Target mRNA expression was measured using TaqMan assays (Thermo Fisher Scientific Inc., USA). RT‐qPCR was performed as described previously [23]. Briefly, cDNA formulated from total RNA by reverse transcription was submitted for qPCR using a TaqMan® Fast Advanced Master Mix kit (Thermo Fisher Scientific Baltics, Lithuania; at 50°C for 2 min, then 95°C for 20 s, followed by 40 cycles of 1‐s denaturation at 95°C and 20‐s annealing/extension at 60°C). GAPDH was used as an internal control, for normalization of PCR results. Gene expression was quantified using the 2^−ΔΔCT^ method. Assays were conducted in duplicate to confirm reproducibility. Information on the TaqMan primer sequences for apolipoprotein A1 (APOA1, assay ID: Cf02682463), ATP‐binding cassette subfamily B member 4 (ABCB4, assay ID: Cf02647380), Glyceraldehyde‐3‐phosphate dehydrogenase (GAPDH, assay ID: Cf04419463) is available at: https://www.thermofisher.com/order/genome-database/.

### 2.5 Functional and Pathway Enrichment Analysis

The Database for Annotation Visualization and Integrated Discovery (DAVID; https://davidbioinformatics.nih.gov/) online tool was used to conduct Gene Ontology (GO), and Kyoto Encyclopedia of Genes and Genomes (KEGG) pathway enrichment analyses were used to elucidate the functions and biological significance of identified DEGs in canine HCC [28]. GO terms comprised biological process (BP), cellular component (CC), and molecular function (MF). The dog genome (*Canis lupus familiaris*) was selected as the background parameter. *p*‐values < 0.05 were regarded as statistically significant in both analyses. Cytoscape v3.10.3 software (Cytoscape Consortium, San Diego, USA, https://cytoscape.org/) was used to visualize networks for enriched pathways.

### 2.6 Protein‐Protein Interaction Networks, Hub Genes and Module Analysis

A protein‐protein interaction (PPI) network was constructed, targeting proteins encoded by DEGs with expression value > 100 TPM, using the Search Tool for the Retrieval of Interacting Genes/Proteins (STRING v12.0; https://www.string-db.org/) database [32]. PPI networks with confidence score ≥ 0.4 were then visualized using Cytoscape v3.10.3 [32]. A Molecular Complex Detection (MCODE) plug‐in [28] was applied to determine significant modules in the network, with the following criteria: degree cutoff = 2, *k*‐score = 2, node score cutoff = 0.2, and max. depth = 100. Module enrichment analysis was conducted using the online DAVID database [28]. The 10 most highly expressed hub genes in each PPI network were ascertained with Cytoscape's Maximal Clique Centrality (MCC) algorithm, and visualized using the CytoHubba plug‐in [33].

### 2.7 Cross‐Species Transcriptomic Analysis

Canine HCC data were compared with human HCC datasets from the GEO database, including GSE183250 (tissue samples comprising both intrahepatic and metastatic HCC; HCC: *n* = 18; tumor‐adjacent normal liver: *n* = 18; https://www.ncbi.nlm.nih.gov/geo/query/acc.cgi?acc=GSE183250) and GSE203329 (cell line samples: *n* = 12, only three wild‐type RNA‐seq Huh‐7‐cell datasets were included; https://www.ncbi.nlm.nih.gov/geo/query/acc.cgi). Data were downloaded directly to CLC Genomics Workbench v24.0, and the reads were processed to identify DEGS in human HCC. DEGs in human HCC were determined by mapping to human genome reference assembly GRCh38/hg38 in CLC Workbench. DEGs common to canine and human HCC were identified using Venn diagrams. To reduce potential bias arising from differences in sequencing platforms, sample handling, or analytical pipelines between datasets, both canine and human RNA‐seq data were processed using a harmonized workflow with consistent filtering thresholds, normalization strategies, and DEG criteria. Only one‐to‐one orthologous genes were included in cross‐species analyses to ensure comparability.

### 2.8 Survival Analysis and Gene Expression of Key Candidate Genes

Key human‐canine orthologous genes were assessed for prognostic value in human HCC using Kaplan‐Meier (KM) plotter (https://kmplot.com/analysis/) and The Encyclopedia of RNA Interactomes (ENCORI, https://rnasysu.com/encori/) databases [34]. Patients were grouped by median gene expression, with *p* < 0.05 considered as statistically significant. Genes significant in both databases were further validated for expression in human HCC using the Gene Expression Profiling Interactive Analysis 2 (GEPIA2, http://gepia.cancer-pku.cn/), based on TCGA (The Cancer Genome Atlas) and GTEx data.

### 2.9 Statistical Analysis

Canine HCC and normal liver qPCR expression data were compared using the Mann–Whitney *U*‐test. Chromosomes harboring statistically significantly high numbers of upregulated or downregulated genes were identified with Fisher's exact test. Associations between expression patterns of DEGs common to canine and human HCC were evaluated using Spearman's rank correlation coefficient. Statistical significance was defined as *p* < 0.05, with *p* < 0.01 was considered highly significant (*p < 0.05, **p < 0.01, ***p < 0.001). All statistical analyses and visualizations were performed using GraphPad Prism v10.0 (San Diego, USA) (https://www.graphpad.com/).

### 2.10 Use of Artificial Intelligence Tools

To improve the linguistic quality of this manuscript, we employed ChatGPT‐4o (OpenAI) for English grammar correction and expression refinement. All AI‐assisted outputs were carefully reviewed, verified, and revised by the authors to ensure accuracy and appropriateness. No content related to study design, data analysis, results interpretation, or conclusions was generated by artificial intelligence.

## 3 Results

### 3.1 RNA‐Seq Reveals Robust Read Quality and Distinct Transcriptomic Profiles in Canine HCC

NGS analysis yielded 70 to 81 million paired‐end reads (2 × 100 bp reads; Phred score > 36) for the six targeted samples (Table S2), with high alignment rates versus the reference genome (mean ± SD: 93.18 ± 1.25% for HCC, 91.30 ± 2.78% for normal liver; Figure S1A), and distinct separation in PCA (Figure S1B). Four of the most highly expressed genes in canine HCC were unique to healthy liver tissues, and a further 11 showed altered rankings (Figure S1C), indicating sufficient depth for differential expression analysis. Together, these results confirm the high quality and reliability of the RNA‐seq data, supporting its suitability for subsequent differential expression and downstream bioinformatic analyses.

### 3.2 Differential Expression Analysis Highlights Novel DEGs and Chromosome 9 and 1 Enrichment in Canine HCC

**Figure 1:**
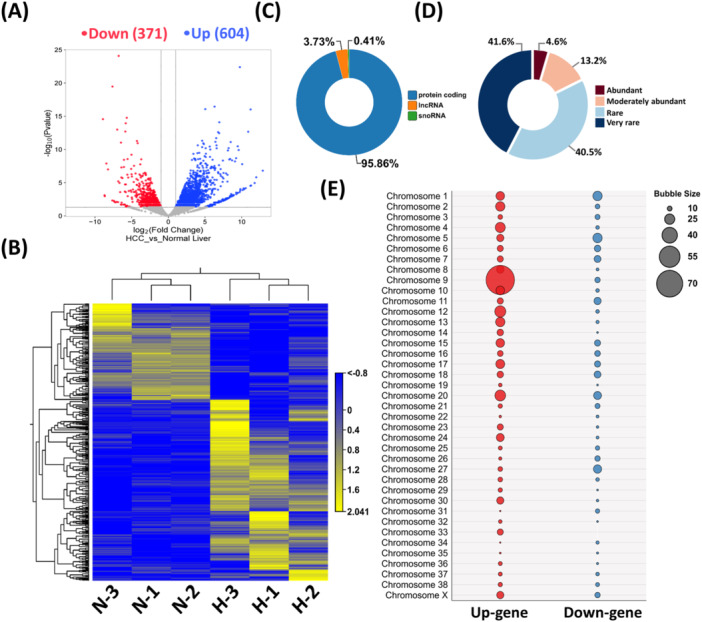
Identification of differentially expressed genes (DEGs) from RNA‐seq data and their chromosomal location. (A) Volcano plot showing the DEGs from RNA‐seq. Each dot represents a gene. Blue dots represent upregulated genes, red dots represent downregulated genes, and gray dots represent genes that were not differentially expressed. DEGs were filtered out using the criteria as follows: *p* < 0.05, |logFC | > 1.5, and max group mean > 5 (expression value). The x‐axis indicates the log2 fold change, and the y‐axis indicates the ‐log10 (*p*‐value) of DEGs. (B) Hierarchical clustering of HCC and normal liver samples based on gene expression. H‐HCC, N‐Normal liver. (C) Biotypes of the DEGs in canine HCC (D) Expression abundance of DEGs. Expression values (maximum group mean) of the DEGs were classified as very rare (5–15), rare (16–99), moderately abundant (100–499), and abundant (> 500). (E) Bubble plot showing the chromosomal locations of the DEGs. Here, the bubble size represents the number of DEGs located on each chromosome.

To scan for aberrantly expressed genes in canine HCC, we submitted HCC and healthy liver tissues to NGS analysis. We identified 975 DEGs (604 upregulated and 371 downregulated; Table S3), as visualized in volcano plots and hierarchical clustering (Figure 1A, B). Notably, 146 DEGs (83 upregulated, 63 downregulated) were annotated as novel by Ensembl, lacking UniProtKB/Swiss‐Prot or RefSeq matches (Table S4). The ten most upregulated DEGs were AFP, SPP1, ACTG1, GNAS, ABCB4, PI3, SDC1, NRBP2, MTSS1, and FABP1 (Table 1), and the ten most downregulated were ALB_1, DHODH, APOA1, ALDOB, APOC1, APOC3, CRP, SAA1_2, HPD, and SERPINA3 (Table 2). DEGs were categorized according to their maximum group mean expression values as very rare (5–15), rare (16–99), moderately abundant (100–499), and abundant ( > 500), following the approach described earlier [23]. For downstream analyses, including PPI network construction and hub gene identification, only moderately abundant and abundant DEGs were considered, as these are more likely to have biologically significant roles. Identified DEGs (935/975) commonly coded proteins (Figure 1C) and were mainly classified as very rarely (*n* = 406, 41.64%) or rarely (*n* = 395, 40.51%) expressed (Figure 1D). Approximately 18% of DEGs (*n* = 174) exhibited high expression (max group mean ≥ 100), classified as moderately abundant or abundant (Figure 1D). Upregulated and downregulated DEGs were significantly clustered on chromosome 9 (*n* = 79; *p* < 0.0001, OR: 4.5 [95% CI: 2.4–8.5], RR: 1.46 [95% CI: 1.3–1.5]) and chromosome 1 (*n* = 24; *p* < 0.05, OR: 1.9 [95% CI: 1.03–3.5], RR: 1.43 [95% CI: 1.04–1.8]), respectively (Figure 1E; Table S5). Read alignment revealed region‐specific peak gain or loss in these chromosomes between healthy liver and HCC tissues (Figure S2).

**Table 1:** Maximally up‐regulated DEGs (1–20) in canine HCC. DEGs are ranked based on their expression level. Other criteria for selection of up‐regulated DEGs were: |logFC | > 1.5 and *p* < 0.05.

Gene	ENSEMBL ID	Chromosome	Max group mean	Log₂ fold change	*p‐*value	FDR *p‐*value
AFP	ENSCAFG00845005947	13	2288.28	11.22	9.56E‐17	2.68E‐13
gene:ENSCAFG00845010871	ENSCAFG00845010871	3	2164.64	2.82	1.95E‐04	7.26E‐03
SPP1	ENSCAFG00845016131	32	2160.33	5.5	3.56E‐07	5.29E‐05
ACTG1	ENSCAFG00845010637	9	1880.58	1.52	4.00E‐02	2.70E‐01
GNAS	ENSCAFG00845013733	24	828.5	2.11	5.40E‐03	8.00E‐02
ABCB4	ENSCAFG00845007730	14	715.93	2.1	5.55E‐03	8.00E‐02
PI3	ENSCAFG00845014356	24	565.89	7.13	9.06E‐08	1.66E‐05
SDC1	ENSCAFG00845018675	17	479.69	1.5	4.00E‐02	2.80E‐01
NRBP2	ENSCAFG00845005761	13	465.4	1.74	3.00E‐02	2.60E‐01
MTSS1	ENSCAFG00845011807	13	419.1	1.69	4.00E‐02	2.80E‐01
FABP1	ENSCAFG00845013057	17	390.52	4.15	8.36E‐05	3.85E‐03
PTGR1	ENSCAFG00845005537	11	349.72	2.68	2.36E‐05	1.40E‐03
TIMP1	ENSCAFG00845030572	X	342.29	3.12	3.97E‐04	1.00E‐02
STEAP3	ENSCAFG00845028945	19	334.71	3.19	1.61E‐04	6.37E‐03
NADK2	ENSCAFG00845004315	4	287.18	2.01	2.00E‐02	1.60E‐01
NQO1	ENSCAFG00845009149	5	273.8	2.46	1.23E‐04	5.20E‐03
TSPO	ENSCAFG00845014235	10	259.4	1.68	1.96E‐03	4.00E‐02
ANXA2	ENSCAFG00845017171	30	258.76	2.94	3.81E‐05	2.00E‐03
ABCB11	ENSCAFG00845020713	36	253.07	1.54	3.00E‐02	2.30E‐01
CCL26	ENSCAFG00845004038	6	251.25	10.89	2.14E‐13	2.77E‐10

Abbreviations: DEGs, Differentially expressed genes; FDR, False discovery rate; HCC, Hepatocellular carcinoma; logFC, Log₂ fold change.

**Table 2:** Maximally down‐regulated DEGs (1–20) in canine HCC. DEGs are ranked based on their expression level. Other criteria for selection of down‐regulated DEGs are: |logFC | > 1.5 and *p* < 0.05.

Gene	ENSEMBL ID	Chromosome	Max group mean	Log₂ fold change	*p‐*value	FDR *p‐*value
ALB_1	ENSCAFG00845005754	13	110,752.98	−1.52	4.00E‐02	2.90E‐01
DHODH	ENSCAFG00845007968	5	21,868.25	−3.6	7.21E‐06	5.59E‐04
APOA1	ENSCAFG00845004266	5	20,056.51	−2.03	5.95E‐03	9.00E‐02
ALDOB	ENSCAFG00845009188	11	17,297.97	−2.07	2.00E‐02	1.60E‐01
APOC1	ENSCAFG00845006666	1	8746.04	−1.5	4.00E‐02	2.80E‐01
APOC3	ENSCAFG00845004284	5	8610.71	−3.57	1.07E‐06	1.18E‐04
CRP	ENSCAFG00845030990	38	5577.45	−2.33	4.00E‐02	2.70E‐01
SAA1_2	ENSCAFG00845015494	21	4618.34	−6.4	1.62E‐06	1.64E‐04
HPD	ENSCAFG00845023604	26	3987.24	−1.57	3.00E‐02	2.40E‐01
SERPINA3	ENSCAFG00845003163	8	3975.35	−2.8	4.71E‐03	7.00E‐02
SAA1_1	ENSCAFG00845015397	21	3753.84	−5.8	1.83E‐05	1.15E‐03
HAMP	ENSCAFG00845003240	1	3235.43	−6.46	9.22E‐12	7.38E‐09
CDO1	ENSCAFG00845005340	11	2548.70	−2.18	1.69E‐03	4.00E‐02
CAT	ENSCAFG00845024098	18	2391.86	−1.82	2.00E‐02	1.70E‐01
MT2A	ENSCAFG00845003852	2	2,277.59	−1.81	3.00E‐02	2.30E‐01
C1R	ENSCAFG00845025120	27	1705.66	−1.74	2.00E‐02	2.00E‐01
SERPINF2	ENSCAFG00845014866	9	1439.69	−1.84	1.00E‐02	1.60E‐01
APOA5	ENSCAFG00845004306	5	1361.06	−3.46	8.70E‐05	3.96E‐03
SERPINF1	ENSCAFG00845013723	9	1313.10	−2.14	6.37E‐03	9.00E‐02
XBP1	ENSCAFG00845030211	26	1251.70	−2.09	4.25E‐03	7.00E‐02

Abbreviations: DEGs, Differentially expressed genes; FDR ‐ False discovery rate; HCC, Hepatocellular carcinoma;logFC, Log₂ fold change.

### 3.3 RT‐qPCR Validation Confirms RNA‐Seq–Derived Differential Expression Patterns

**Figure 2:**
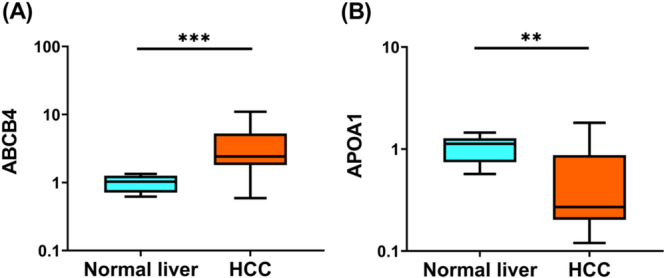
The expression levels of ABCB4 and APOA1 in normal liver tissues and canine HCC clinical tissues. (A) Box and whiskers plot representing the relative expression of ABCB4 in canine HCC tissues (normal liver tissues, *n* = 6; HCC, *n* = 16) and (B) Box and whiskers plot representing the relative expression of APOA1 in canine HCC tissues (normal liver tissues, *n* = 6; HCC, *n* = 16). Statistical differences between normal liver and HCC tissues were evaluated using the Mann–Whitney *U* test. The y‐axis represents the relative expression level of respective mRNAs normalized with GAPDH in log10 units. **p < 0.01, ***p < 0.001.

To verify the RNA sequencing results from NGS, we validated expression for one upregulated (ABCB4) and one downregulated (APOA1) DEG by RT‐qPCR and confirmed consistent expression patterns (upregulated ABCB4 and downregulated APOA1; Figure 2A,B).

### 3.4 Canine HCC Exhibits Enriched Oncogenic Pathways and Metabolic Pathway Suppression Based on DEG Profiling

**Figure 3:**
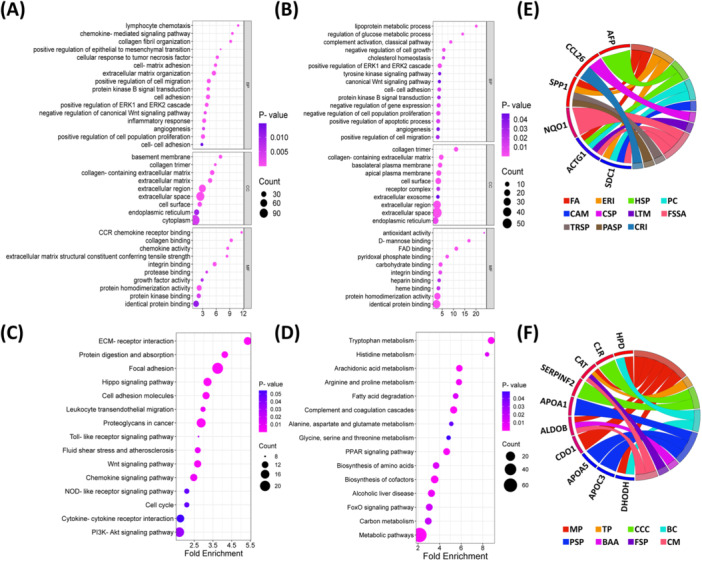
Gene Ontology and KEGG pathway analysis of the differentially expressed genes (DEGs) in canine HCC. (A–B) Gene Ontology analysis of upregulated DEGs (A) and downregulated DEGs (B). GO terms include significant terms in biological process (BP), cellular component (CC), and molecular function (MF). (C–D) Enriched KEGG Pathways of upregulated DEGs (C) and inhibited pathways of downregulated DEGs (D). The KEGG pathways and GO terms are listed on the left, while *p*‐values and gene counts are shown on the right. (E–F) Chord diagram representing the association between KEGG pathways and top 20 upregulated (E) and downregulated (F). BAA, biosynthesis of amino acids; BC, biosynthesis of cofactors; CAM, cell adhesion molecules; CCC, complement and coagulation cascades; CM, carbon metabolism; CRI, cytokine–cytokine receptor interaction; CSP, chemokine signaling pathway; DEGs, differentially expressed genes; ERI, ECM–receptor interaction; FA, focal adhesion; FSP, FoxO signaling pathway; FSSA, Fluid shear stress and atherosclerosis; HSP, hippo signaling pathway; LTM, leukocyte transendothelial migration; MP, metabolic pathways; PASP, PI3K/Akt signaling pathway; PC, proteoglycans in cancer; PSP, PPAR signaling pathway; TP, tryptophan metabolism; TRSP, toll‐like receptor signaling pathway.

To explore transcriptomic regulation in canine HCC, we targeted all DEGs in GO and KEGG enrichment analyses. For upregulated DEGs (*n* = 604), significantly enriched (*p* < 0.05) GO terms were biological processes (*n* = 86), cellular components (*n* = 33), and molecular functions (*n* = 29) [Table S6], with chemokine‐signaling, ECM (extracellular matrix) organization, positive regulation of (epithelial‐mesenchymal transition), ERK cascades, cell‐matrix adhesion, cell migration, proliferation, and angiogenesis as key enriched terms (Figure 3A). For downregulated DGEs (*n* = 371), significantly enriched GO terms were biological processes (*n* = 56), cellular components (*n* = 19), and molecular functions (*n* = 19), with ERK cascades, tyrosine kinase, and Wnt signaling, cell adhesion, proliferation, migration, apoptosis, angiogenesis, and glucose metabolism (Figure 3B) as key enriched terms. Notably, DEG sets were highly associated with the extracellular space and ECM dysregulation, key drivers of tumor progression.

KEGG analysis revealed 39 and 20 significant (*p* < 0.05) pathways (Table S7) for upregulated and downregulated DEGs, respectively. For upregulated DEGs, key enriched pathways were ECM‐receptor interaction, focal adhesion, proteoglycans in cancer, Wnt signaling, chemokine signaling, and PI3K/Akt signaling (Figure 3C). Contrastingly, downregulated DEGs were mainly associated with carbon, amino acids, and lipid metabolism, PPAR signaling, complement and coagulation cascades, FoxO signaling, and biosynthesis of amino acids and cofactors (Figure 3D). Among the top dysregulated DEGs, three upregulated genes (SPP1, ACTG1, and SDC1) interacted with at least four KEGG pathways (Figure 3E), while 5 downregulated genes displayed multiple pathway interactions (Figure 3F).

### 3.5 PPI Network Analysis Reveals ACTG1 as the Most Connected Node Among DEGs

**Figure 4:**
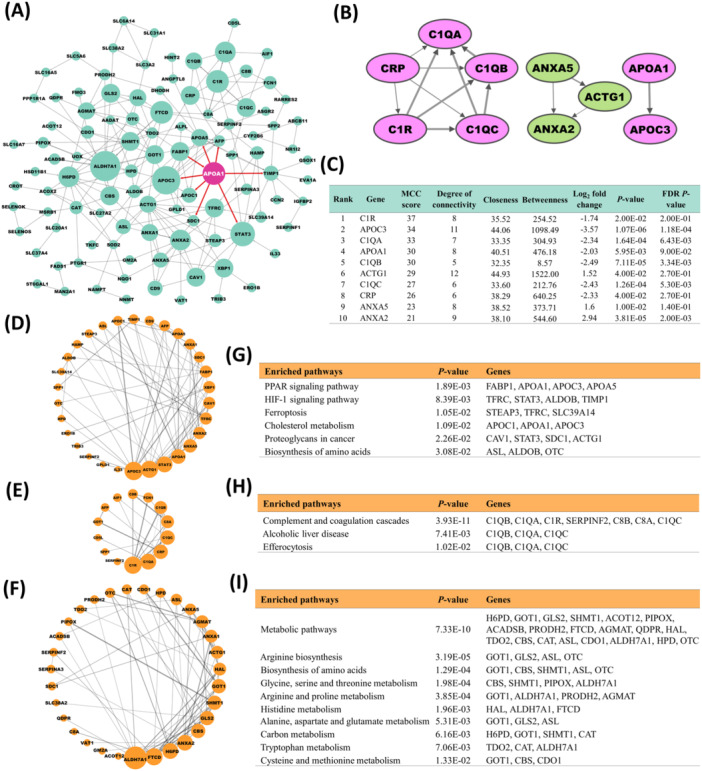
Protein–protein interaction (PPI) network, hub gene identification, and module analysis from the DEGs in canine HCC. (A) PPI networks of the 174 differentially expressed mRNAs, consisting of 196 edges and 146 nodes. Here, pink node indicates the validated downregulated gene (APOA1) in the present study, while the upregulated gene (ABCB4) did not show interaction with other genes. The PPI network was constructed via Cytoscape v3.10.3. The size of the node indicates the degree of connectivity of a specific gene in the PPI network. (B) The PPI network of the top 10 hub genes, visualized by Cytoscape. The up and downregulated genes are shown by a color gradient from green to pink, respectively. (C) Detailed information on the top 10 hub genes in the PPI network. (D–F) Top 3 modules of the PPI network: module 1 with 30 nodes (D), module 2 with 14 nodes (E), and module 3 with 31 nodes (F). (G–I) KEGG pathways, exhibited by module 1 (G), module 2 (H), and module 3 (I). KEGG pathways were formulated using DAVID bioinformatics.

To elucidate biological pathways or processes impacted by canine HCC, we constructed a PPI network for proteins encoded by highly expressed DEGs (51 upregulated and 123 downregulated DEGs, classified as moderately abundant or abundantly expressed; Table S8). The resultant PPI network comprised 146 nodes and 196 edges (Figure 4A). In this network, the 10 maximally expressed hub genes included ACTG1, ANXA5, and ANXA2 (upregulated), and C1R, APOC3, C1QA, APOA1, C1QB, C1QC, and CRP (downregulated) [Figure 4B]. In evaluation of hub gene properties (Figure 4C), ACTG1 showed the highest (*n* = 12) degree of connectivity.

### 3.6 Module Analysis Identifies Three Major Functional Gene Clusters and Localizes Key Hub Genes in Canine HCC

In the PPI network, seven modules were generated, and the three most significant (MCODE scores ≥ 3.9 and nodes ≥ 14) are presented in Figure 4D–F. The DEGs in the three most significant PPI‐network (Figure 4D–F) modules (*n* = 61) included all 10 hub genes (Figure S3A), with ACTG1, ANXA2, and ANXA5 participating in modules 1 and 3 (Figure S3B). Module 1 (score: 4.552) contained 30 nodes and 66 edges (Figure 4D), module 2 (score: 4) comprised 14 nodes and 26 edges (Figure 4E), and module 3 (score: 3.933) comprised 31 nodes and 59 edges (Figure 4F). Pathway enrichment annotation revealed modules 1 and 3 were closely related (nine shared genes, and metabolism‐related pathways in both models; Figure 4G,I and S3B). Additionally, PPAR signaling, HIF‐1 signaling, and proteoglycans in cancer were other significant (*p* < 0.05) KEGG pathways exhibited by module 1 (Figure 4G). The DEGs in module 2 encoded complement and coagulation cascades, alcoholic liver disease, and efferocytosis pathways (Figure 4H).

### 3.7 Comparative RNA‐Seq Highlights Conserved Oncogenic Signatures between Canine and Human HCC

**Figure 5:**
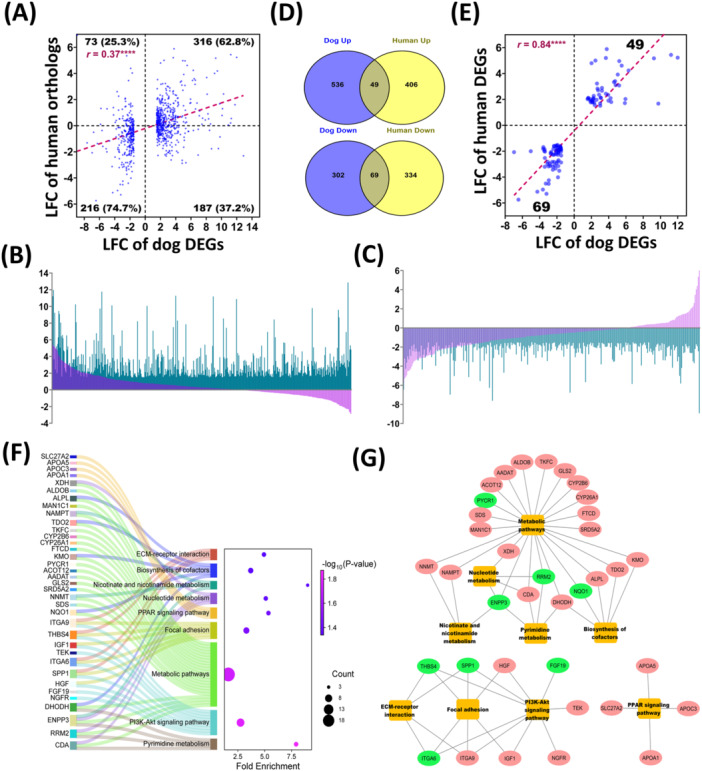
Cross‐species analysis of differentially expressed genes (DEGs) between canine and human HCC. (A) Scatter plot showing the correlation of log_2_ fold change (LFC) of 792 similar genes in canine DEGs (horizontal axis) and human GEO dataset (vertical axis). Spearman correlation analysis showed a weak positive (*r* = 0.37) and a highly significant (****p < 0.0001) relationship between the species. (B‐C) Bar diagram showing the overlaps of the LFC values for 792 common genes, with 503 upregulated DEGs (B) and 289 downregulated DEGs (C) in canine HCC. Here, green represents the dog DEGs, and pink for human genes. (D) Venn diagram showing the overlap of DEGs between the species, identifying 49 common upregulated and 69 downregulated DEGs. (E) Scatter plot showing the correlation of LFC values of 118 shared DEGs between canine (horizontal axis) and human (vertical axis) HCC. Spearman correlation analysis showed a strong positive (*r* = 0.84) and a highly significant (****p < 0.0001) relationship between the species. (F) KEGG pathway analysis of 118 commonly shared DEGs using DAVID bioinformatics. The identified KEGG pathways and their associated genes are listed on the left, while *p*‐values and gene counts are displayed on the right. (G) Network visualization of enriched pathways and their corresponding genes using Cytoscape v3.10.3. Here, yellow nodes represent KEGG pathways, while green and red for upregulated and downregulated genes respectively.

The human HCC RNA‐seq dataset (GSE183250) yielded 455 upregulated and 403 downregulated DEGs after mapping against human genome reference assembly (GRCh38/hg38) using criteria similar to the canine NGS dataset (Table S9). The DEGs are visualized in a volcano plot, PCA, and hierarchical clustering (Figure S4). Of 975 canine DEGs, 792 had human orthologs. Cross‐species analysis showed that 63% (*n* = 316) of upregulated and 75% (*n* = 216) of downregulated orthologs had consistent fold‐change directions. Overall, orthologous gene expression showed a significant, weak positive correlation between species (*r* = 0.37, *p* < 0.0001), illustrated in scatter and bar plots (Figure 5A‐C). Focusing on the genes specifically identified as DEGs, we identified 49 commonly upregulated ( ~ 8% and 11% of the respective canine and human DEGs) and 69 commonly downregulated DEGs (~ 19% and 17% respectively; Figure 5D). These 118 shared DGEs showed a strong, significant positive correlation in expression (*r* = 0.84; *p* < 0.0001; Figure 5E; Table S10), highlighting substantial cross‐species overlap.

In hierarchical clustering, all orthologous genes (*n* = 14,899) expressed in canine and human HCC tissues were clustered together, and human HCC cell‐line (GSE203329) genes were clustered separately (Figure S5A). In PCA plots, human HCC DEGs were closer to canine equivalents, than to the cell‐line dataset (Figure S5B).

### 3.8 Cross‐Species Pathway Analysis Reveals Conserved Oncogenic and Metabolic Pathways in Canine and Human HCC

KEGG pathway analysis of 118 human–canine orthologous DEGs (49 upregulated and 69 downregulated) identified nine shared pathways, with pyrimidine metabolism, PI3K/Akt signaling, and metabolic pathways being the most significant (Figure 5F; Table S11). Other enriched pathways included focal adhesion, PPAR signaling, and ECM‐receptor interaction, with downregulated genes dominating most pathways (Figure 5G). Several genes including SPP1, THBS4, ITGA6, ENPP3, RRM2, NQO1, DHODH, CDA, ITGA9, HGF, IGF1, KMO, TDO2, ALPL, XDH, NAMPT, and NNMT were involved in multiple pathways (Figure 5F,G). DAVID pathway analysis revealed 36 of 118 orthologous were enriched in cross‐species pathways. Notably, SPP1, NQO1 (upregulated), and APOA1, APOC3, APOA5, DHODH, ALDOB (downregulated) ranked among the top expressed DEGs in canine HCC.

### 3.9 Prognostic Evaluation Reveals SPP1, NQO1, RRM2, APOA1, ALDOB, and IGF1 as Survival Indicators

We evaluated the key orthologous genes identified in KEGG pathway analysis as prognosticators of survival for human HCC using ENCORI and the Kaplan–Meier plotter database. Poorer overall survival was predicted by higher SPP1, NQO1, and RRM2 expression, and lower APOA1, APOC3, ALDOB, and IGF1 expression (Figures S6–S9). All three upregulated (SPP1, NQO1, and RRM2) and three of four downregulated genes (APOA1, ALDOB, and IGF1) showed significant (*p* < 0.05) differential expression in human HCC when compared to normal liver tissues via GEPIA2 (Figure S10), demonstrating consistency across canine and human datasets.

## 4 Discussion

Here, we report on aberrant gene expression in canine HCC, a potential spontaneous model of human HCC. To the best of our knowledge, this is the most comprehensive transcriptome analysis of canine HCC to date, covering DEG‐related pathways and chromosomal location, and the first comparative evaluation of dysregulation patterns in canine and human HCC.

The 975 DEGs in canine HCC identified in this study showed the greatest clustering on chromosome 9 (upregulated DEGs) or chromosome 1 (downregulated DEGs), indicating that these two chromosomes may harbor genes crucial for HCC development and progression. Three of the 10 maximally downregulated DEGs in canine HCC were apolipoproteins (APOA1, APOC1, and APOC3) known to be prognosticators in human HCC [35].

Dysregulation was of a smaller magnitude for upregulated than downregulated DEGs, but some findings for the former are of oncological interest. AFP is a well‐known HCC biomarker [36], while SPP1 (osteopontin) is a potential diagnostic, prognostic, and therapeutic marker involved in tumor progression, apoptosis inhibition, migration, invasion, angiogenesis, and immune suppression [37, 38, 39, 40], and its silencing reportedly inhibits HCC growth and metastasis both in vitro and in vivo, with accompanying modulation of tumor‐associated apoptosis resistance [41]. ACTG1, ABCB4, and SDC1 have reported roles in human HCC [42, 43]. However, the role of these upregulated DEGs in canine HCC remains unclear.

Ontologically, most DEG‐encoded proteins were located in the cytoplasm and extracellular space. They may play key roles in the ECM, a key component of the tumor microenvironment linked to cancer progression through tumor cell proliferation, survival, and migration [44]. Other identified GO terms included cell adhesion, cell‐matrix adhesion, collagen fibril organization, ECM organization and integrin binding, which represent closely interconnected processes that potentially influence ECM structure and function [45]. ECM‐immune cell interactions within the tumor microenvironment reportedly drive cancer metastasis [46], which is consistent with the ontological enrichment of ECM and immune‐related terms reported here (chemokine‐mediated signaling pathway, CCR chemokine receptor binding, and lymphocyte chemotaxis).

Upregulated DEGs were enriched in pathways such as ECM‐receptor interaction, focal adhesion, cell cycle, proteoglycans, PI3K/Akt, and Wnt signaling, all of which are well‐established drivers of tumor progression in both humans and dogs [23, 28, 30, 45, 47, 48]. These pathways collectively promote proliferation, survival, migration, and angiogenesis, which are the key hallmarks of HCC. The extracellular matrix (ECM), comprising collagens, elastin, proteoglycans, and glycoproteins not only provides structural support but also regulates essential cellular processes, including adhesion, migration, differentiation, survival, and proliferation [49]. SPP1 and SDC1 are known key ECM‐receptor genes [50], and SPP1 reportedly promotes HCC via PI3K/Akt/mTOR signaling [51]. Osteopontin (SPP1) is a phosphorylated glycoprotein that is abundantly expressed in chronic inflammatory liver diseases, including HCC. Moreover, its level is markedly elevated in the plasma of patients with clinical HCC, making it a valuable diagnostic biomarker. Functionally, SPP1 promotes tumor cell proliferation, migration, and metastasis by activating MAPK, NF‐κB, and PI3K/Akt signaling pathways [52]. Human HCC frequently exhibits activation of the Wnt/β‐catenin signaling pathway (reported in up to 50% of cases) and the PI3K/Akt pathway (40%–60%) [53, 54], as well as ECM‐associated pathways. The Wnt/β‐catenin cascade regulates key cellular processes, including tumor initiation, growth, survival, migration, differentiation, and apoptosis. Mutations in β‐catenin are closely linked to disease prognosis and have emerged as promising targets for novel molecular therapeutic strategies [54]. Again, the activation of immune‐related pathways (cytokine signaling, NOD‐like receptor signaling, chemokine signaling, toll‐like receptor signaling, and leukocyte migration) in canine HCC highlights their possible roles in tumor proliferation, EMT, immune evasion, and metastasis in human HCC [55]. Their enrichment in canine HCC implies a similarly remodeled tumor microenvironment characterized by chronic inflammatory signaling and impaired antitumor immunity. Accordingly, we consider that the strong extracellular localization of DEGs in the extracellular space (based on the GO term) and significant enrichment of ECM‐associated pathways in this study strongly implicate SPP1 and other ECM‐related proteins in canine HCC progression.

Contrastingly, downregulated DEGs were mainly associated with suppressed metabolic pathways, indicating significant metabolic reprogramming favoring cancer growth. This shift, exemplified by the Warburg effect, leads to enhanced glycolysis to meet increased energy demands despite the presence of oxygen, thereby reducing reliance on oxidative pathways such as fatty acid degradation and amino acid metabolism [56]. Hypoxia in tumor microenvironments may further suppress fatty acid and cofactor metabolism, while reduced carbon metabolism suggests impairment in gluconeogenesis and other hepatocyte functions. Decreased tryptophan and arginine metabolism may contribute to immune evasion as they play potential role in immune modulation [57, 58]. Notably, the PPAR signaling pathway, essential for lipid homeostasis, was also suppressed. APOA1, a key PPAR regulator [59], was among the most downregulated genes, potentially promoting a lipid‐rich environment for tumor growth. Loss of PPAR function may further enhance inflammation and immune modulation [60], as reflected by upregulated cytokine and chemokine pathways. Thus, restoring APOA1 expression using mimetics may provide a potential therapeutic approach for HCC. Overall, the enrichment of ECM remodeling and Wnt/β‐catenin signaling pathways supported by metabolic and immune‐modulatory alterations illustrates their coordinated impact on the tumor microenvironment and the advancement of HCC.

In this study, we performed a comprehensive cross‐species transcriptomic analysis to evaluate molecular similarities between canine (NGS datasets from the present study) and human (GEO datasets) HCC. The findings support the utility of companion dogs as a biologically relevant model for human liver cancer. Unlike induced rodent models, dogs develop HCC spontaneously within an intact immune system. They are naturally exposed to environmental and lifestyle factors [7] comparable to those of humans, and their cancers thus more accurately reflect the complexity and heterogeneity of human malignancies than rodent tumors. Furthermore, the high genomic and molecular homology between dogs and humans, as reported previously [8], together with the substantial overlap in dysregulated genes and enriched pathways identified in our analysis, reinforces the strong translational potential of canine HCC as a comparative model for advancing diagnostic and therapeutic strategies in human HCC. Notably, in PCA plots, and hierarchical clustering, canine and human HCC showed greater transcriptomic similarity with each other than with a human HCC cell line. Furthermore, canine HCC DEGs and human orthologs were dysregulated in the same direction in 63% (upregulated) or 75% (downregulated) of cases. The further identification of 49 upregulated and 69 downregulated DEGs as orthologous supports the possibility of conserved oncogenic (and thus molecular) functions for canine and human HCC. Importantly, the shared DEGs were associated with several conserved genes and pathways, including SPP1, NQO1, ACTG1, ALDOB, and IGF1, which may hold potential as diagnostic markers or therapeutic targets between the species. Additionally, the significant involvement of DEGs in ECM‐receptor interaction and other ECM‐related pathways supports the view that ECM remodeling promotes cancer cell growth and survival and enhances metabolic reprogramming [61] in both species. Collectively, the identification of these shared molecular signatures and conserved oncogenic pathways strengthens the translational significance of our findings and underscores the utility of canine HCC as a comparative model for biomarker discovery and therapeutic development relevant to human HCC.

This study has several limitations. First, the sample size was relatively small, and tumor‐adjacent normal liver tissue was not available. Additionally, the control samples were not matched to the clinical HCC cases by age, sex, or breed. Although RT‐qPCR validation in an independent cohort of clinically diverse HCC cases supported the reliability of our transcriptomic findings, the study primarily included spontaneous canine HCC cases and lacked stratification by underlying etiology. Future investigations incorporating different etiological subtypes, larger and well‐balanced cohorts are required to evaluate the influence of breed, age, and sex on gene expression. Second, although the human dataset GSE183250 includes both intrahepatic and distant metastatic HCC samples, all available tumor samples were collectively analyzed for cross‐species comparison against normal liver tissues. We acknowledge that subgroup analysis based on metastatic status was not performed due to sample constraints, which may limit stage‐specific interpretation. Third, only two DEGs were validated through RT‐qPCR due to limited experimental resources. Comprehensive validation of additional candidate genes and associated pathways, including confirmation at the protein level, will be essential to strengthen the translational relevance of these findings. Finally, cross‐species comparative analyses have inherent constraints, as canine and human datasets have been generated independently and differ in sequencing platform, and biological context such as genetic background, tumor heterogeneity, etiological factors, and species‐specific immune or metabolic conditions. Although a uniform analysis pipeline and one‐to‐one ortholog mapping were applied to minimize these effects, future studies with larger and well‐matched datasets are necessary to confirm the biomarker potential and clinical significance of the identified targets and to advance comparative oncology research.

In conclusion, we identified DEGs likely to play critical roles in HCC progression in dogs, with APOA1, APOC3, CRP, AFP, SPP1, ACTG1, and ANXA2 as highly dysregulated hub genes, and thus potential targets in future biomarker and therapeutic target investigations. We suggest that ECM remodeling together with metabolic reprogramming and immune evasion following pathway suppression may play a central role in HCC progression in dogs. Furthermore, the substantial overlap in DEGs between canine and human HCC underscores the value of dogs as a suitable model for studying human HCC.

## Author Contributions

Mohammad Arif and Naoki Miura were involved in conceptualization; Mohammad Arif, MD Nazmul Hasan, Nobuhiro Nozaki, Yutaro Ide and Naoki Miura were involved in Methodology; Mohammad Arif, MD Nazmul Hasan, Nobuhiro Nozaki, Yutaro Ide, Yoshiyuki Akiyama, Shaohsu Wang, Most Shumi Akhter Shathi, Osamu Yamato and Naoki Miura were involved in resources; Mohammad Arif, MD Nazmul Hasan, Nobuhiro Nozaki, Yutaro Ide, Yoshiyuki Akiyama, Shaohsu Wang, Most Shumi Akhter Shathi and Osamu Yamato were involved in data curation; Mohammad Arif and Most Shumi Akhter Shathi were involved in investigation; Mohammad Arif was involved in formal analysis, visualization, and writing original draft; Naoki Miura was involved in supervision, funding acquisition, project administration, manuscript review and editing. All authors have read and approved the final version of the manuscript.

## Ethics Statement

This study was approved by the Ethics Committee of Kagoshima University (Approval No. KVH220001). All procedures involved patient handling were conducted in accordance with the guidelines of the Ethics Committee.

## Consent

This study does not contain personal information. Written informed consent for sample collection was obtained from all patients’ owners prior to the surgery.

## Conflicts of Interest

The authors declare no conflicts of interest.

## Supporting information


**Figure S1:** Characterization of RNA‑Seq reads from canine HCC.

**Figure S2:** Reads mapped from each individual sample to chromosomes 1 and 9 in dog HCC RNA‐Seq data. **Figure S3:** Assessment of similarities in differentially expressed genes (DEGs) among different modules identified from the protein‐protein interaction (PPI) network of canine HCC. **Figure S4:** Identification of differentially expressed genes (DEGs) from human HCC RNA‐Seq data (GEO dataset: GSE183250; HCC tissue: *n* = 18, tumor‐adjacent healthy liver tissue: *n* = 18). **Figure S5:** Cross‐species similarities in gene expression profiles. **Figure S6:** Survival analysis of six upregulated differentially expressed genes common to canine and human HCC using the KM plotter online database. **Figure S7:** Survival analysis of six upregulated differentially expressed genes common to canine and human HCC using the ENCORI online database. **Figure S8:** Survival analysis of 15 downregulated differentially expressed genes common to canine and human HCC using the KM plotter online database. **Figure S9:** Survival analysis of 15 downregulated differentially expressed genes common to canine and human HCC using the ENCORI online database. **Figure S10:** Expression analysis for key candidate genes in canine and human HCC using GEPIA2 online database. **Table S1:** Overview of samples included in the study. **Table S2:** Sample‐wise description of total reads, mapped and unmapped reads and their percentage in canine HCC RNA‐Seq data. **Table S3:** Identification of differentially expressed genes (DEGs) in canine HCC. **Table S4:** A list of novel genes identified in canine HCC RNA‐Seq data. **Table S5:** Chromosomal distribution of upregulated and downregulated differentially expressed genes (DEGs) in canine HCC. **Table S6:** A list of all GO terms identified from the upregulated and downregulated differentially expressed genes (DEGs) in canine HCC using DAVID bioinformatics. **Table S7:** A list of all KEGG pathways identified from the upregulated and downregulated differentially expressed genes (DEGs) in canine HCC using DAVID bioinformatics. **Table S8:** A list of 51 upregulated and 123 downregulated differentially expressed genes (DEGs) in canine HCC targeted in protein‐protein interaction (PPI) network. **Table S9:** Identification of differentially expressed genes (DEGs) in human HCC. **Table S10:** Differentially expressed genes (DEGs) common to canine and human HCC. **Table S11:** KEGG pathways identified in canine and human HCC from commonly shared DEGs between the two species.

## Data Availability

Raw RNA‐seq data of human HCC (GSE183250) and human HCC cell lines (GSE203329) were obtained from the Gene Expression Omnibus (GEO) database. The canine HCC datasets generated in this study are available in the NCBI BioProject database under accession number PRJNA1293103 (https://www.ncbi.nlm.nih.gov/bioproject/?term=PRJNA1293103). Other data are available within the article and the supporting materials.
