# Supplementary material for: Molecular Insights Into Canine Hepatocellular Carcinoma: A Cross‐Species Transcriptomic Comparison With Human HCC
Source: Mol Carcinog. 2026 Feb 10;65(4):523–36. doi: 10.1002/mc.70092 (PMC12973160; doi:10.1002/mc.70092)
Supplement: Supplementary file 1 — Figure S1: Characterization of RNA‑Seq reads from canine HCC. [file MC-65-523-s001.docx]

**Molecular insights into canine hepatocellular carcinoma: A cross-species transcriptomic comparison with human HCC**

Mohammad Arif^1,2^, MD Nazmul Hasan^1^, Nobuhiro Nozaki^1^, Yutaro Ide^1^, Yoshiyuki Akiyama¹, Shaohsu Wang^1^, Most Shumi Akhter Shathi^1^, Osamu Yamato^1^ and Naoki Miura^1,3^

^1^ Joint Graduate School of Veterinary Medicine, Kagoshima University, Kagoshima 890-0065, Japan

^2^ Department of Microbiology and Hygiene, Bangladesh Agricultural University, Mymensingh-2202, Bangladesh

^3^ Veterinary Teaching Hospital, Joint Faculty of Veterinary Medicine, Kagoshima University, Kagoshima 890-0065, Japan

**Correspondence**

Naoki Miura, Joint Graduate School of Veterinary Medicine, Kagoshima University, Japan

Tel.: +81-99-285-3527

E-mail: [k9236024@kadai.jp](mailto:k9236024@kadai.jp)

**Supplementary Figures**


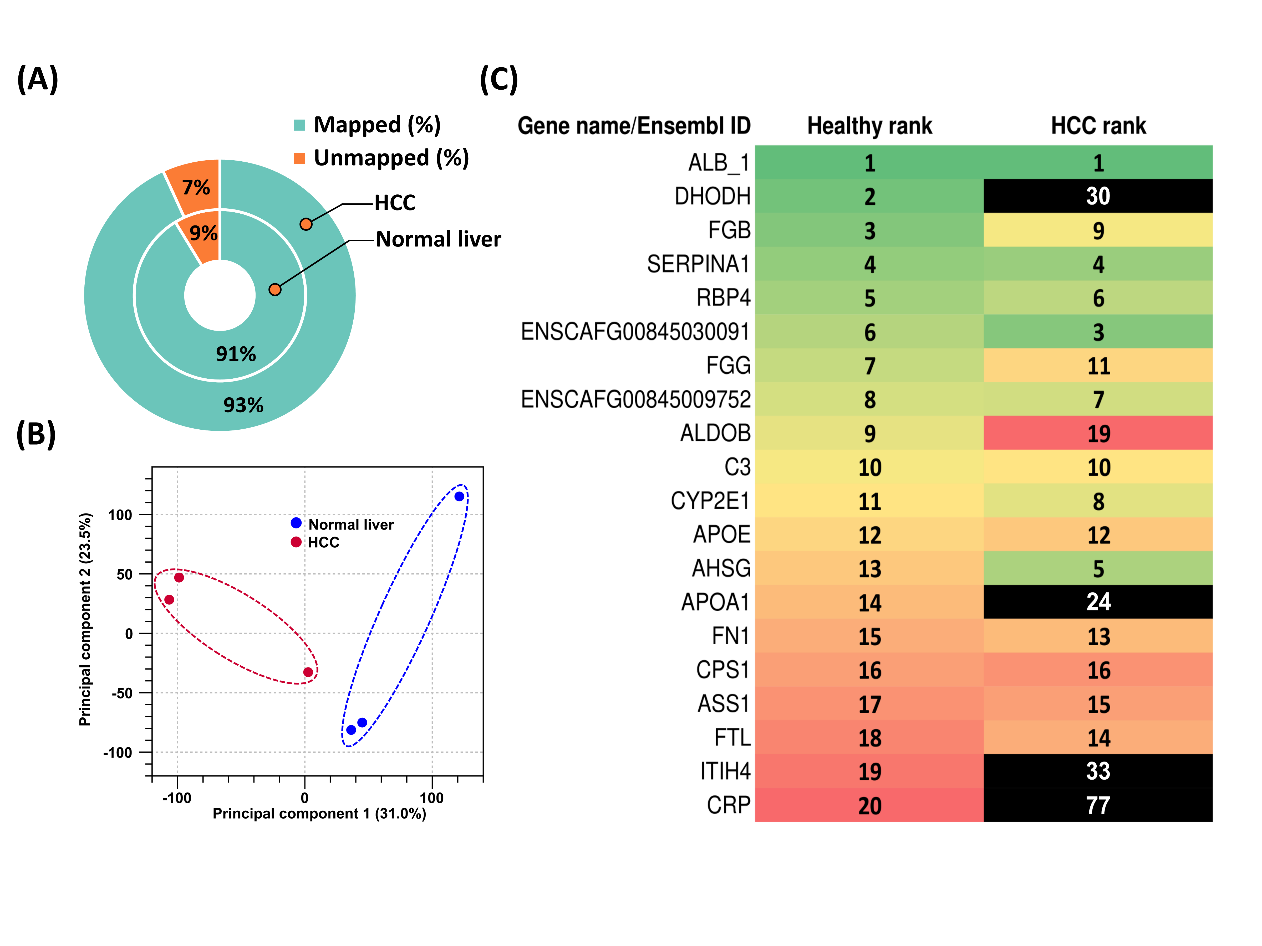


**Figure S1.** Characterization of RNA‑Seq reads from canine HCC (**A**) Average percentage of mapped reads against the reference genome in each group; normal liver, n = 3; HCC, n = 3. (**B**) Principal component analysis (PCA) plot between HCC and normal liver from the whole transcriptome by CLC Workbench v24.0. The axis indicates the variance contribution. Blue is for Normal liver and red is for HCC samples. (**C**) Comparison of top twenty expressed genes in normal liver and their relative rank in HCC. Numbers and color gradient (green to red) indicate highest to lowest ranking. Genes not ranked in the top 20 HCC group were marked in black color.


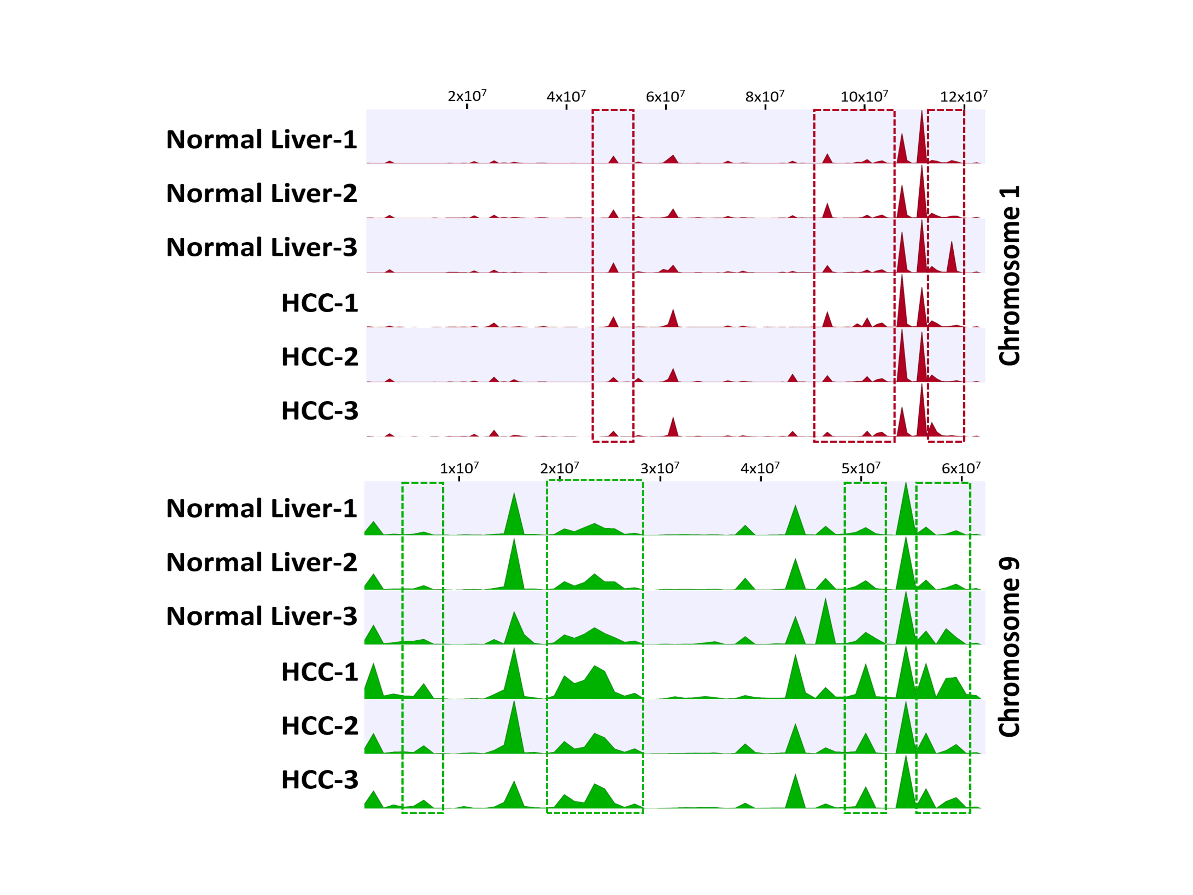


**Figure S2.** Reads mapped from each individual sample to chromosomes 1 and 9 in dog HCC RNA-Seq data. Dotted lines indicate variations in mapped sequences between groups. The x-axis represents chromosome length, while the y-axis represents the number of mapped sequences for each sample.


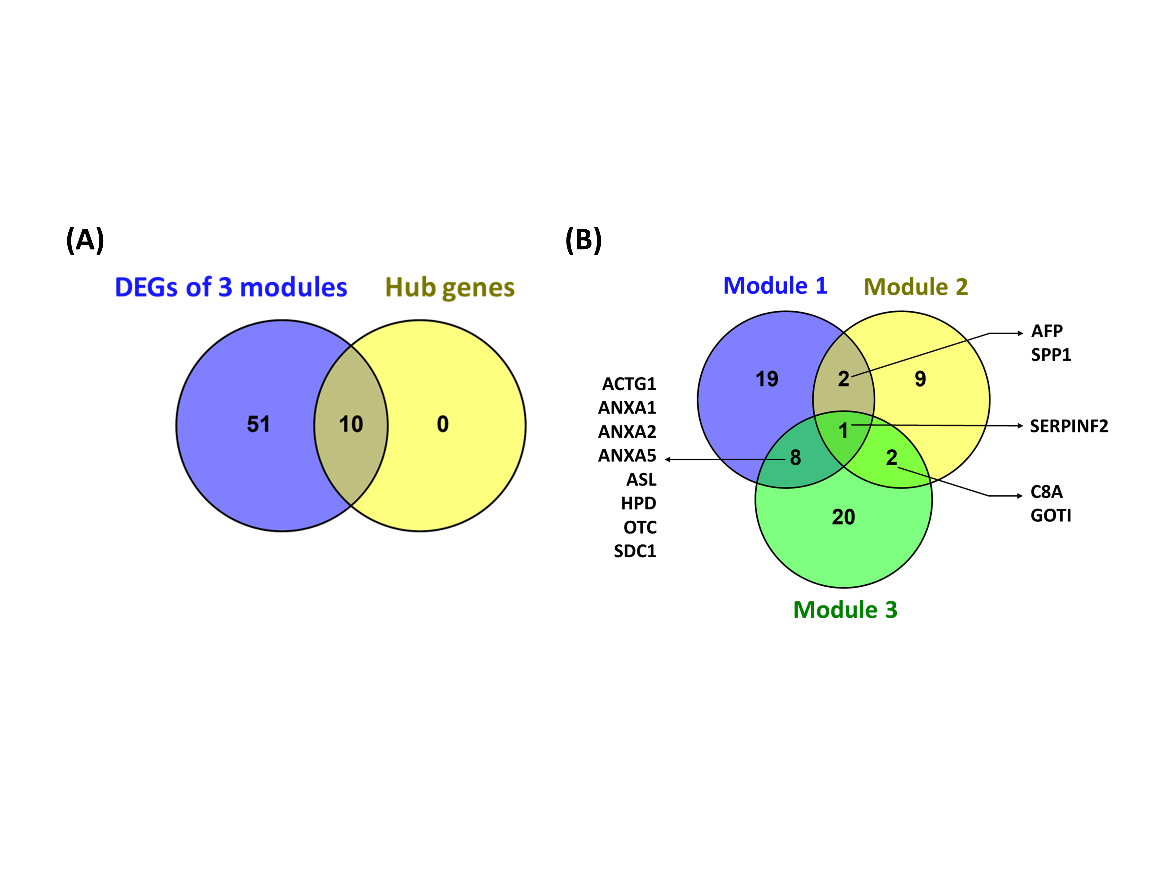


**Figure S3.** Assessment of similarities in differentially expressed genes (DEGs) among different modules identified from the protein-protein interaction (PPI) network of canine HCC (**A**) Venn diagram showing common DEGs between hub genes and the top three modules of the PPI network. (**B**) Venn diagram showing common DEGs among the top three modules of the PPI network.


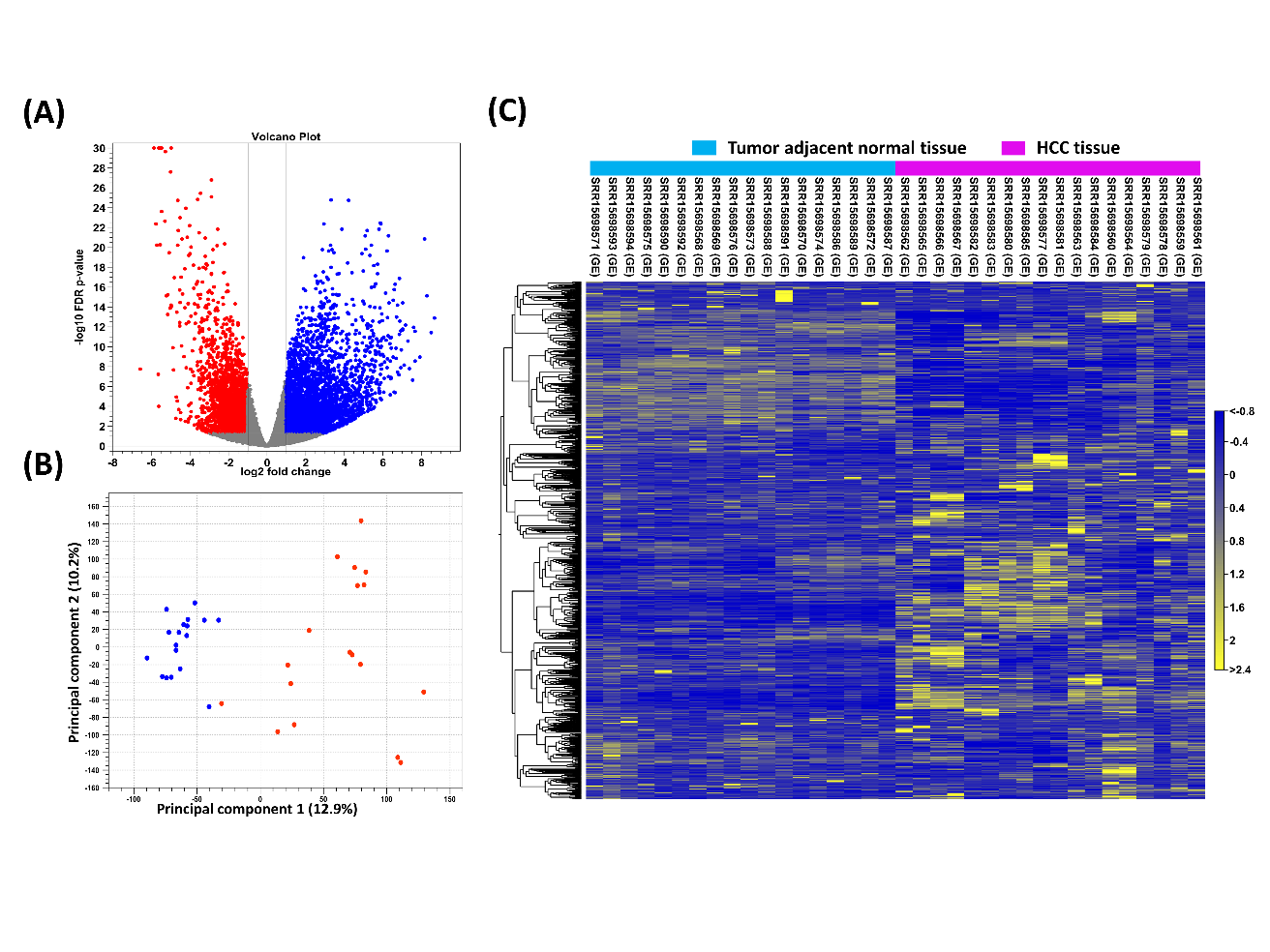


**Figure S4.** Identification of differentially expressed genes (DEGs) from human HCC RNA-Seq data (GEO dataset: GSE183250; HCC tissue: n = 18, tumor-adjacent healthy liver tissue: n = 18) (**A**) Volcano plot showing the DEGs (455 upregulated and 403 downregulated) filtered using the criteria |logFC| > 1.5, *p*-value < 0.05, and max group mean > 5. Each dot represents a gene: blue dots indicate upregulated genes, red dots indicate downregulated genes, and gray dots represent non-differentially expressed genes. The x-axis represents the Log_2_ fold change, and the y-axis represents the -Log10 (*p*-value). (**B**) Principal Component Analysis (PCA) plot comparing HCC tissues and tumor-adjacent normal liver tissues, generated using CLC Workbench v24.0. Blue represents normal liver samples, and red represents HCC tissues. (**C**) Hierarchical clustering of HCC and normal liver samples based on gene expression values of the DEGs. Blue represents normal liver samples, while pink represents HCC tissues.


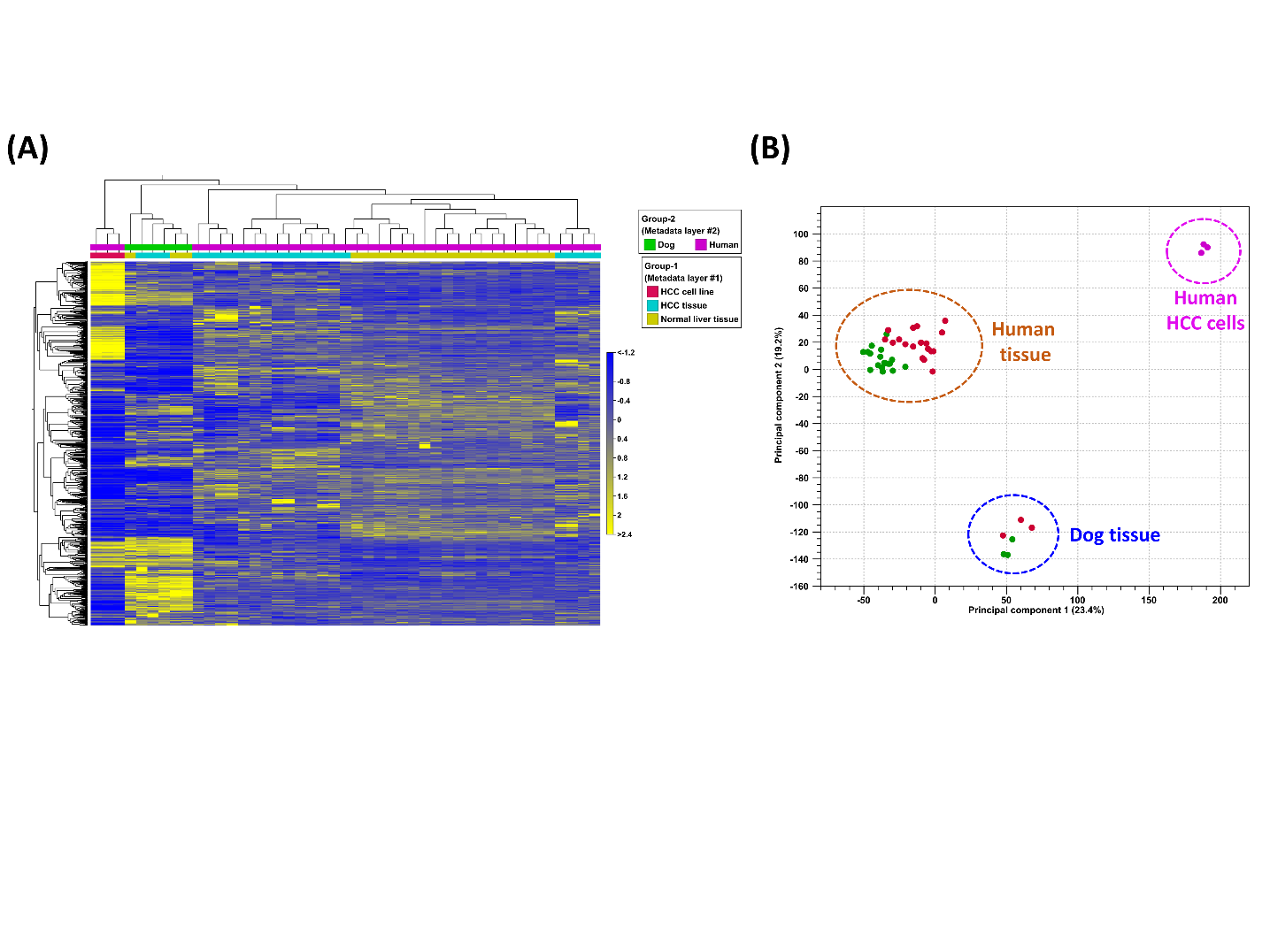


**Figure S5.** Cross-species similarities in gene expression profiles (**A**) Hierarchical clustering of dog and human HCC samples based on a subset of 14,899 genes that are common to dog HCC, human HCC (GSE183250), and the human HCC cell line (GSE203329), without considering DEGs. Each row represents the expression value of a gene across the samples, while each column represents an individual sample. Sample groups are indicated using a color gradient and labeled at the top right corner. (**B**) Principal Component Analysis (PCA) of gene expression profiles from dog HCC, human HCC, and the human HCC cell line, generated using CLC Workbench v24.0. Samples from each species cluster separately: green indicates normal liver tissues, red indicates HCC tissues from both species, and pink denotes human HCC cell line samples. Cluster distance indicates that human HCC shares greater similarity in gene expression profiles with dog HCC than with the human HCC cell line.


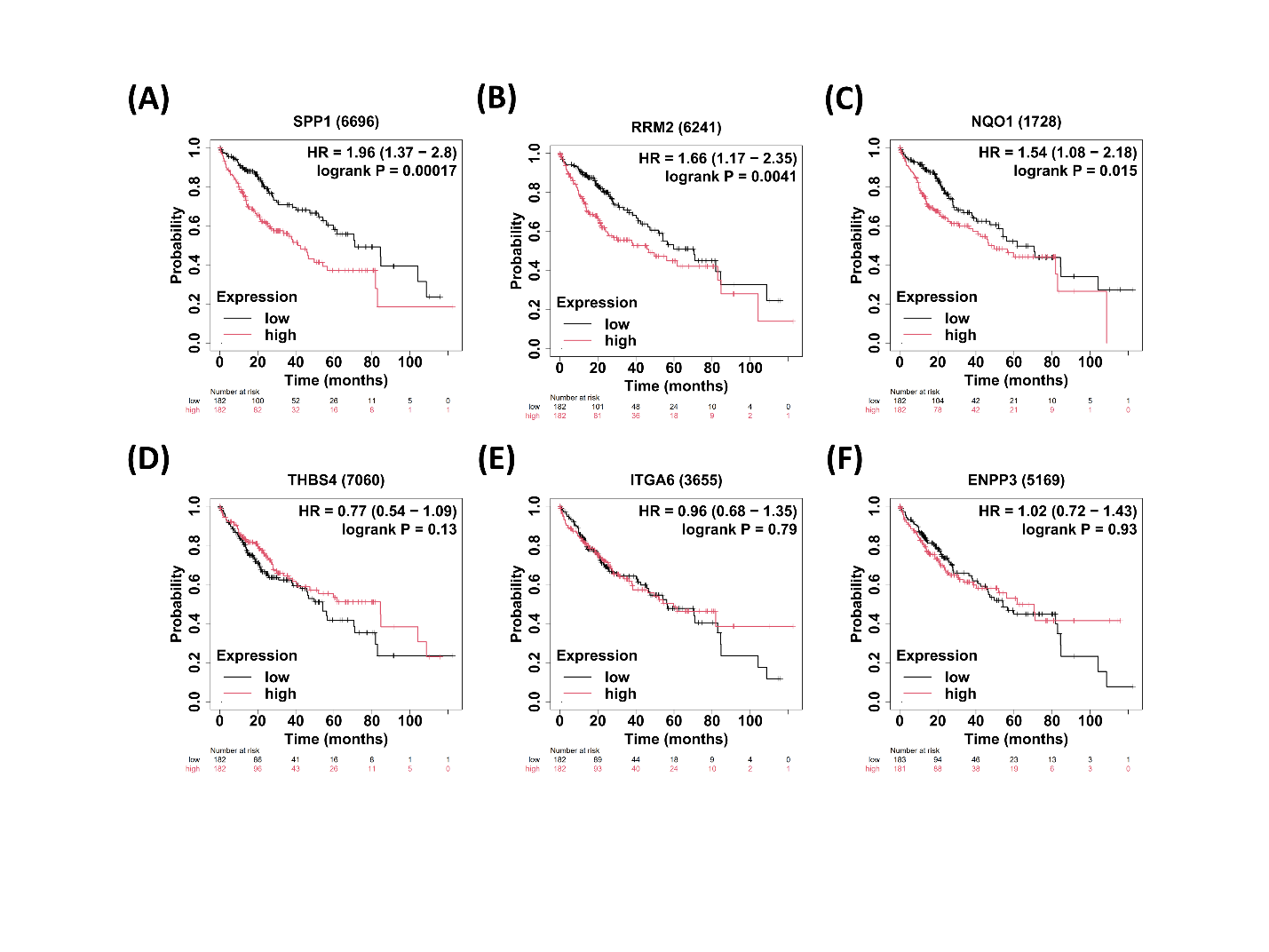


**Figure S6.** Survival analysis of six upregulated differentially expressed genes common to canine and human HCC using the KM plotter online database (**A-F**) Overall survival (OS) analysis of (**A**) SPP1, (**B**) RRM2, (**C**) NQO1, (**D**) THBS4, (**E**) ITGA6, and (**F**) ENPP3 in HCC patients. High expression of SPP1, RRM2, and NQO1 was associated with poorer OS (*p* = 0.00017, 0.0041, and 0.015, respectively), while high expression of THBS4, ITGA6, and ENPP3 was associated with improved OS (*p* = 0.13, 0.79, and 0.93, respectively).


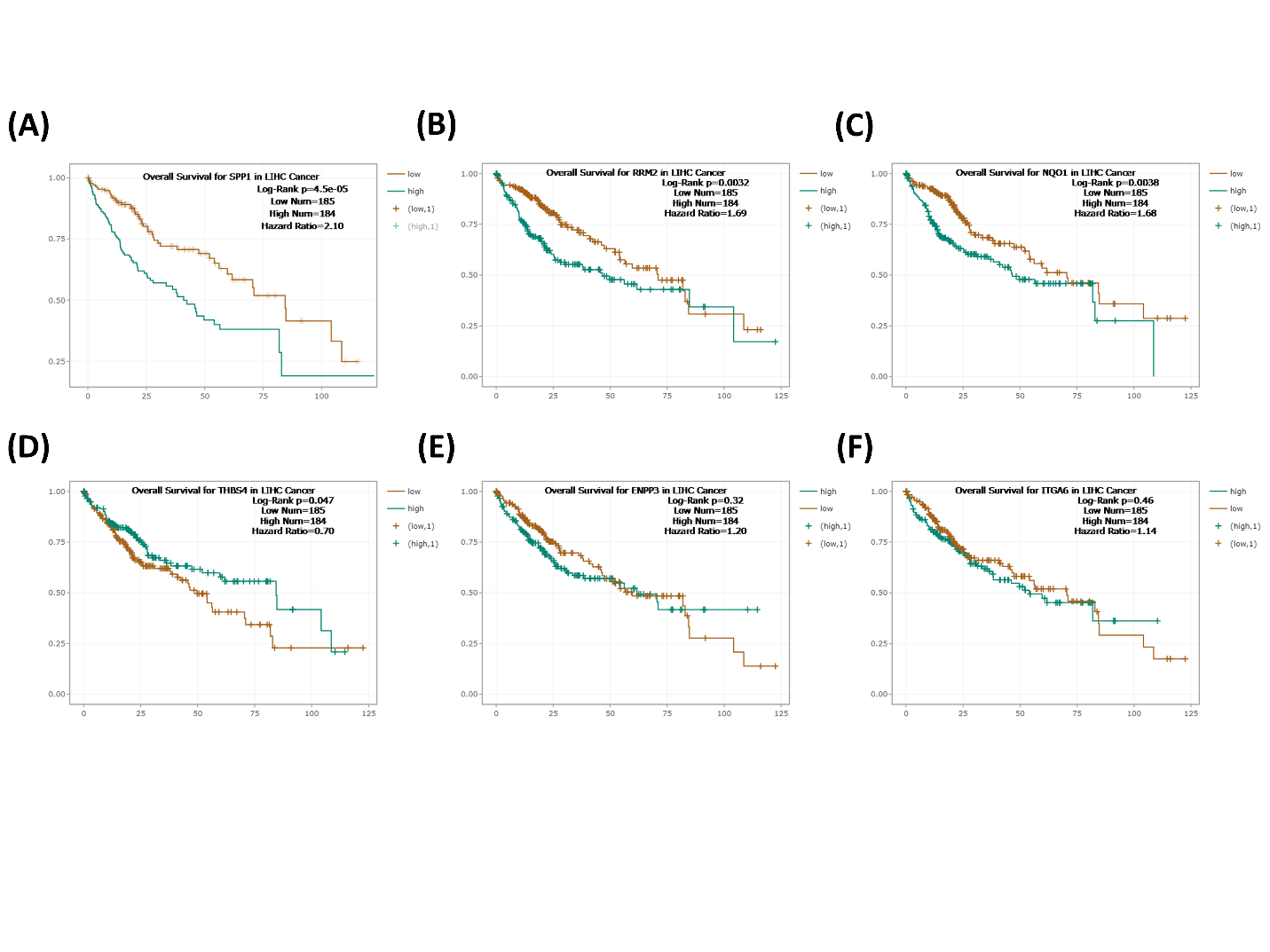


**Figure S7.** Survival analysis of six upregulated differentially expressed genes common to canine and human HCC using the ENCORI online database (**A-F**) Overall survival (OS) analysis of (**A**) SPP1, (**B**) RRM2, (**C**) NQO1, (**D**) THBS4, (**E**) ENPP3, and (**F**) ITGA6 in HCC patients. High expression of SPP1, RRM2, and NQO1 was associated with poorer OS (*p* = 4.5e-05, 0.003, and 0.003, respectively), while high expression of THBS4, ENPP3, and ITGA6 was associated with improved OS (*p* = 0.05, 0.32, and 0.46, respectively).


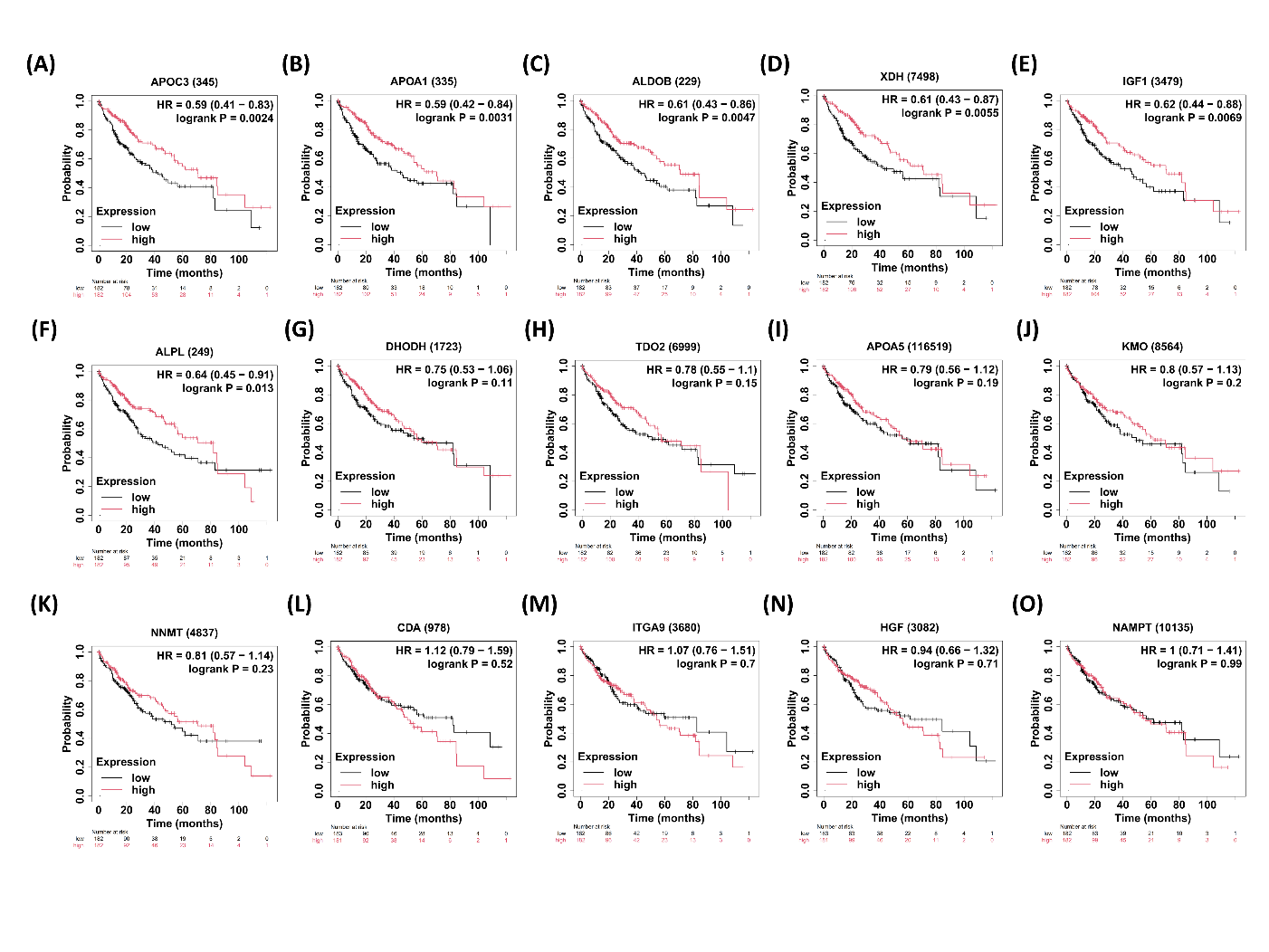


**Figure S8.** Survival analysis of 15 downregulated differentially expressed genes common to canine and human HCC using the KM plotter online database (**A-O**) Overall survival (OS) analysis of (**A**) APOC3, (**B**) APOA1, (**C**) ALDOB, (**D**) XDH, (**E**) IGF1, (**F**) ALPL, (**G**) DHODH, (**H**) TDO2, (**I**) APOA5, (**J**) KMO, (**K**) NNMT, (**L**) CDA, (**M**) ITGA9, (**N**) HGF, and (**O**) NAMPT, in HCC patients.


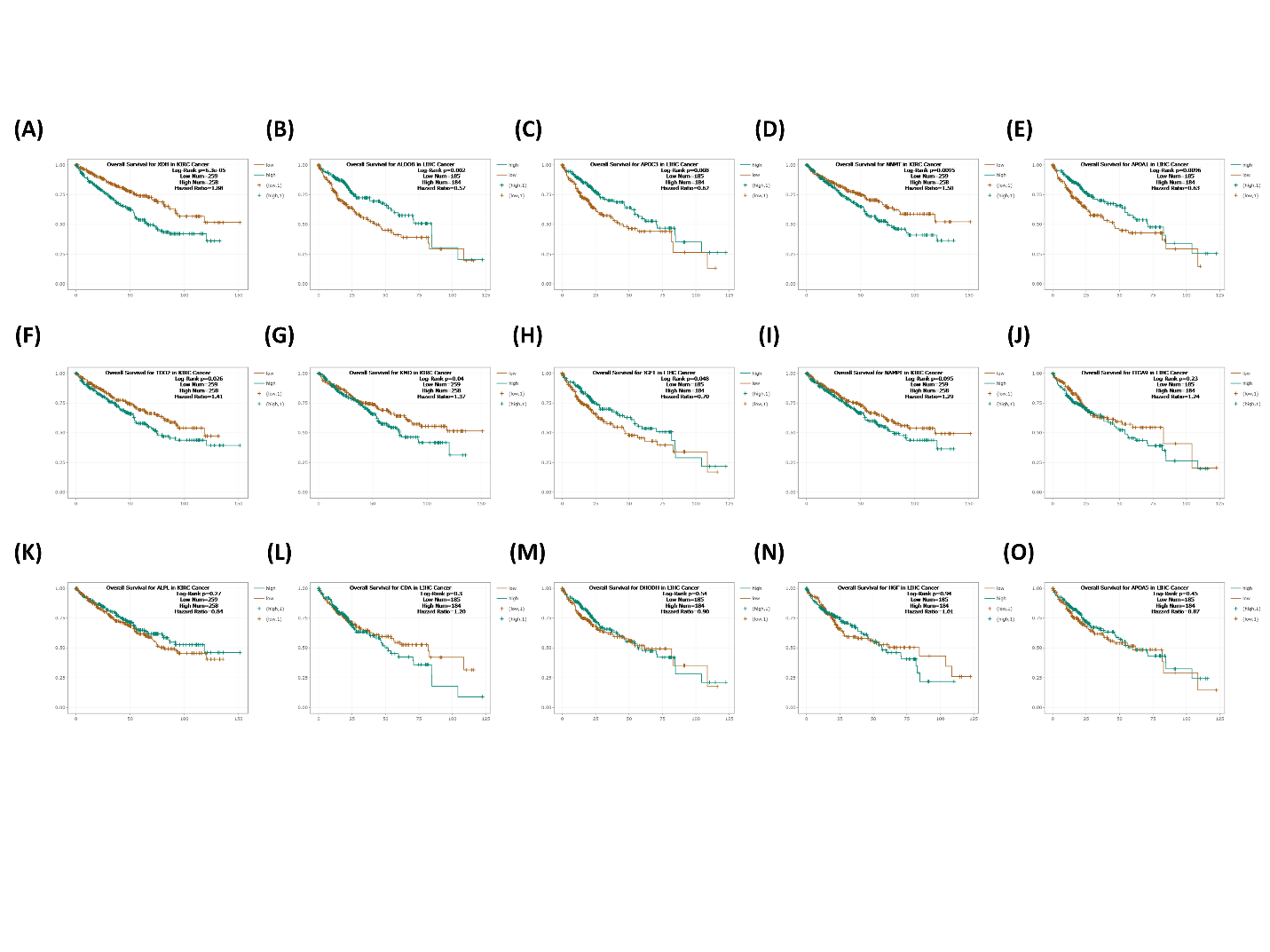


**Figure S9.** Survival analysis of 15 downregulated differentially expressed genes common to canine and human HCC using the ENCORI online database (**A-O**) Overall survival (OS) analysis of (**A**) XDH, (**B**) ALDOB, (**C**) APOC3, (**D**) NNMT, (**E**) APOA1, (**F**) TDO2, (**G**) KMO, (**H**) IGF1, (**I**) NAMPT, (**J**) ITGA9, (**K**) ALPL, (**L**) CDA, (**M**) DHODH, (**N**) HGF, and (**O**) APOA5, in HCC patients.


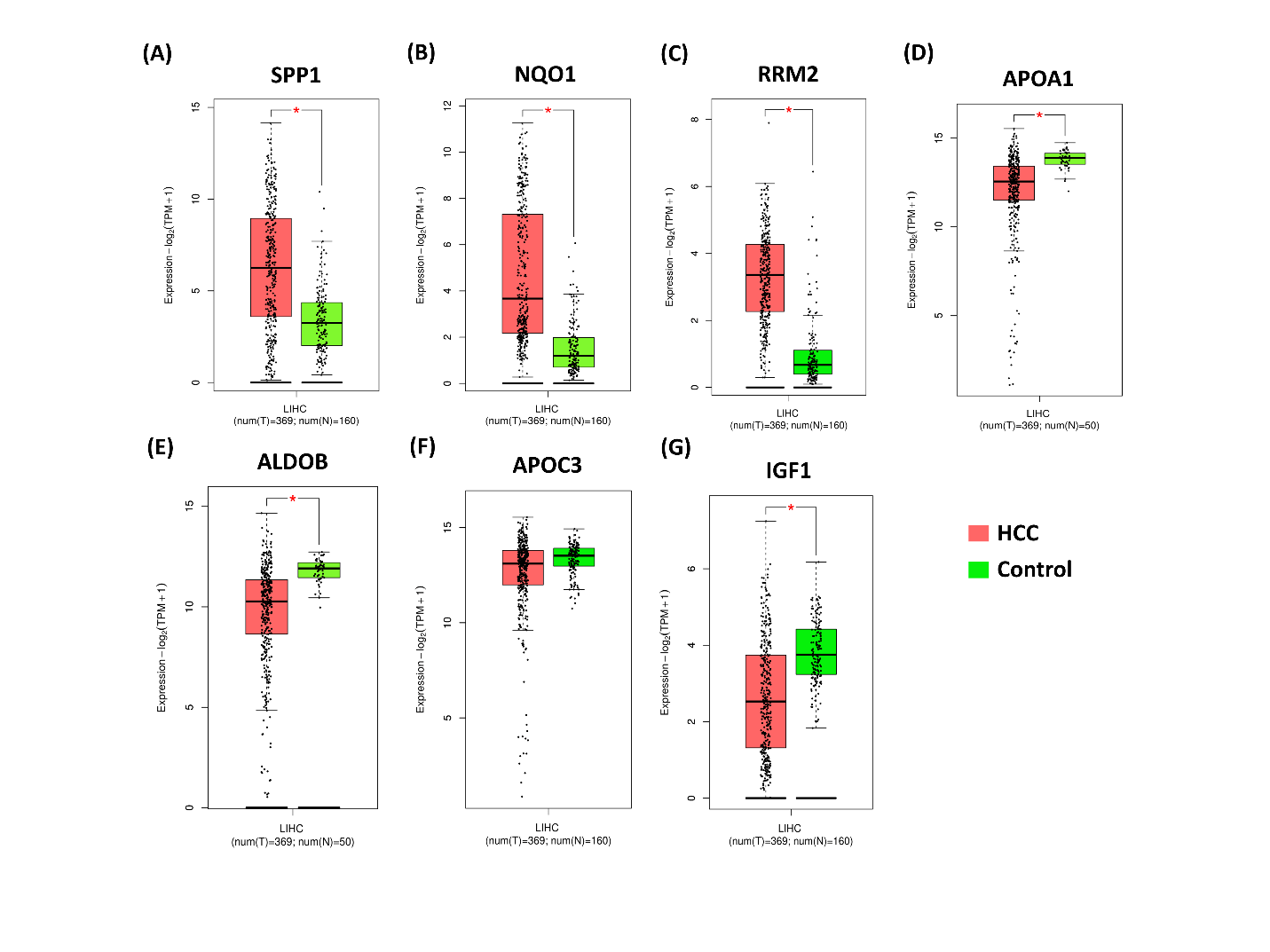


**Figure S10.** Expression analysis for key candidate genes in canine and human HCC using GEPIA2 online database (**A**) SPP1, (**B**) NQO1, (**C**) RRM2, (**D**) APOA1, (**E**) ALDOB, (**F**) APOC3, and (**G**) IGF1. SPP1, NQO1, and RRM2 are overexpressed in HCC tissues while APOA1, ALDOB, APOC3, and IGF1 are downregulated in HCC tissues compared to the normal liver tissues.
